# Learning Then, Learning Now, and Every Second in Between: Lifelong Learning With a Simulated Humanoid Robot

**DOI:** 10.3389/fnbot.2021.669534

**Published:** 2021-07-01

**Authors:** Aleksej Logacjov, Matthias Kerzel, Stefan Wermter

**Affiliations:** Department of Informatics, Research Group Knowledge Technology, Universität Hamburg, Hamburg, Germany

**Keywords:** lifelong learning, self-organizing incremental neural network, growing dual-memory, lifelong learning dataset, simulated humanoid robot, long-term human-robot interaction

## Abstract

Long-term human-robot interaction requires the continuous acquisition of knowledge. This ability is referred to as lifelong learning (LL). LL is a long-standing challenge in machine learning due to catastrophic forgetting, which states that continuously learning from novel experiences leads to a decrease in the performance of previously acquired knowledge. Two recently published LL approaches are the Growing Dual-Memory (GDM) and the Self-organizing Incremental Neural Network+ (SOINN+). Both are growing neural networks that create new neurons in response to novel sensory experiences. The latter approach shows state-of-the-art clustering performance on sequentially available data with low memory requirements regarding the number of nodes. However, classification capabilities are not investigated. Two novel contributions are made in our research paper: (I) An extended SOINN+ approach, called associative SOINN+ (A-SOINN+), is proposed. It adopts two main properties of the GDM model to facilitate classification. (II) A new LL object recognition dataset (v-NICO-World-LL) is presented. It is recorded in a nearly photorealistic virtual environment, where a virtual humanoid robot manipulates 100 different objects belonging to 10 classes. Real-world and artificially created background images, grouped into four different complexity levels, are utilized. The A-SOINN+ reaches similar state-of-the-art classification accuracy results as the best GDM architecture of this work and consists of 30 to 350 times fewer neurons, evaluated on two LL object recognition datasets, the novel v-NICO-World-LL and the well-known CORe50. Furthermore, we observe an approximately 268 times lower training time. These reduced numbers result in lower memory and computational requirements, indicating higher suitability for autonomous social robots with low computational resources to facilitate a more efficient LL during long-term human-robot interactions.

## 1. Introduction

Social robots that interact with humans in their everyday lives are exposed to a dynamic and challenging environment. This dynamic environment provides continuous data streams (Parisi et al., 2019) that are potentially infinite and non-stationary (Ghesmoune et al., 2016; Wiwatcharakoses and Berrar, 2019). Humans can continuously learn throughout their lifespan (Parisi et al., 2019). Hence, to realize an intelligent behavior for long-term human-robot interaction (long-term HRI) in such a complex environment, robots are required to continually acquire new knowledge and adapt to unpredictable changes over time (Dautenhahn, 2004; Parisi et al., 2018; Lomonaco et al., 2019), e.g., by remembering aspects of past interactions with humans (Leite et al., 2013). Current deep learning (DL) approaches, especially convolutional neural networks (CNNs), succeed in many machine learning applications (Najafabadi et al., 2015; Guo et al., 2016). Nevertheless, CNNs rely on a large dataset of (partially) labeled training samples, assuming that all samples are available during training (LeCun et al., 2015; Guo et al., 2016; Parisi et al., 2019). Information that was never seen before is frequently observed in a dynamic environment, requiring a conventional DL approach to be retrained on new and previous observations (Parisi et al., 2018, 2019). This can lead to infeasible memory requirements since all previously observed training samples need to be explicitly stored. The ability to acquire new knowledge over time while preserving previously learned tasks without retraining an architecture from scratch is referred to as *lifelong learning* (LL) (Parisi et al., 2019). LL is a long-standing challenge in machine learning due to *catastrophic forgetting*, which states that continually learning from non-stationary data distributions generally leads to a decrease in the performance of previously learned tasks (Parisi et al., 2019).

Two recently published LL approaches are the *Growing Dual-Memory* (GDM), proposed by Parisi et al. (2018), and the *Self-organizing Incremental Neural Network+* (SOINN+), proposed by Wiwatcharakoses and Berrar (2019). Both are growing neural networks that create new neurons in response to novel sensory experiences. The former model shows state-of-the-art classification and the latter state-of-the-art clustering results on sequentially available data. One of the main differences between these approaches is the utilization of different forgetting strategies. The GDM uses a predefined threshold that defines the maximal age that edges in the network can have before being pruned. If this threshold is too low, catastrophic forgetting can occur since critical units of previously learned tasks can be deleted due to their age (Liew et al., 2019). A too high maximal age can lead to an infeasible amount of neurons. Therefore, an appropriate maximal age must be determined [e.g., by cross-validation (Wiwatcharakoses and Berrar, 2019)], which may not be possible in the real world, with no fixed training data size (Liew et al., 2019). Even though Parisi et al. (2018) state that the periodic replay of neural activation trajectories could be used to prevent the deletion of previously learned knowledge, there was no investigation made concerning the interplay of the maximum age and replay in terms of forgetting. On the other hand, the SOINN+ does not require a predefined maximum age threshold. It calculates this value during the learning process itself. Wiwatcharakoses and Berrar (2019) showed that this forgetting strategy leads to a higher clustering performance and a lower amount of neurons than other growing neural networks. Nevertheless, the authors neither investigated the classification capabilities nor examined the model's behavior on higher than 10-dimensional data.

The promising results of Wiwatcharakoses and Berrar (2019) are the motivation for the development of an extended version of the SOINN+ approach for classification tasks. We make two main contributions. First, we propose two extensions to the SOINN+, adopted from the GDM architecture. (1) An associative matrix is utilized, which stores how often a neuron observed a particular input label, enabling classification. (2) Two additional constraints are introduced that regulate node creation and node adaptation. A new neuron is only created if the input label is unequal to the network's predicted label. On the other hand, nodes are only updated if the input label is equal to the network prediction. We call this extended version associative SOINN+ (A-SOINN+). Second, we present a novel LL object recognition dataset, called v-NICO-World-LL. It exhibits three novelties, to the best of our knowledge, not yet realized in combination by other LL object recognition datasets. (1) The background images are grouped into four different complexity groups, enabling the evaluation of LL models in environments of different levels of complexity. (2) A virtual robot is manipulating objects instead of a human to simulate a long-term HRI scenario where the robot receives different objects over time. (3) It is highly controlled and reproducible, mainly because it is recorded in a nearly photorealistic virtual environment. The dataset consists of 100 different objects, split into 10 categories. Twenty real-world and artificial images are used for the background to simulate different environments. We compare the A-SOINN+ to a conceptually similar state-of-the-art LL approach, the GDM on two LL datasets, the v-NICO-World-LL and the CORe50 (Lomonaco and Maltoni, 2017). Furthermore, we investigate whether the A-SOINN+ can reach state-of-the-art classification accuracy results compared to the GDM while showing fewer memory requirements in terms of created neurons which would be desirable for robots with low computational resources. The A-SOINN+ shows similar high accuracy results as the best GDM model. Simultaneously, a lower amount of units is created, resulting in fewer memory and computational requirements. A further ablation study shows that the new node creation constraint extension leads to the generation of fewer neurons.

Our paper is organized as follows. The following section (Section 2) gives a brief overview of the related work and focuses on the GDM, the SOINN+, and different LL benchmark datasets. The A-SOINN+ approach and the new LL dataset are presented in Section 3. Section 4 shows the experimental setup and the results. The discussion of these results is presented in Section 5. A conclusion and future work are given in Section 6.

## 2. Related Work

### 2.1. Lifelong Learning Approaches

Different approaches try to mitigate catastrophic forgetting in different ways. According to Parisi et al. (2019) and Lesort et al. (2020), those approaches can conceptually be grouped into different categories. This section describes the four categories presented in the work of Lesort et al. (2020), together with example approaches.

#### Dynamic Architecture

Approaches belonging to this category modify the model's architecture dynamically, either explicitly or implicitly. Approaches with explicit architectural modifications create either new models as soon as a new task occurs and connect those models [e.g., Progressive Neural Networks (PNN) (Rusu et al., 2016)] or create new neurons inside the model for each new task, like the *Self-organizing Incremental Neural Network+* (SOINN+) (Wiwatcharakoses and Berrar, 2019) (Subsection 2.3). Approaches that make implicit modifications are not directly changing the architecture. Instead, either a part of the model is deactivated (e.g., freezing weights during backpropagation), or the forward pass paths are modified while learning a new task. Freezing weight approaches are, for example, the hard attention to the task (HAT) (Serrà et al., 2018), the PackNet (Mallya and Lazebnik, 2017), or the Piggyback (PB) (Mallya et al., 2018) approach. The adaptation of the forward pass path is realized in PathNet (Fernando et al., 2017).

#### Regularization

Lesort et al. (2020) present two types of regularization approaches, penalty computing and knowledge distillation. Penalty computing approaches regulate how strong weights are updated while learning a task. Approaches like elastic weight consolidation (EWC) (Kirkpatrick et al., 2016), Synaptic Intelligence (SI) (Zenke et al., 2017), and Memory Aware Synapses (MAS) (Aljundi et al., 2018) search for important weights inside the model and penalize substantial changes to them (Parisi et al., 2019). Hence, the weights are protected from being modified if they are crucial for previously learned tasks (Lesort et al., 2020). Knowledge distillation in a LL context is realized by training a model *A* on task *t*_*i*_. After *A* learned to solve the task, a model *B* is trained to solve a new task *t*_*i*+1_ and to generate the same output as *A*. Hence, knowledge is distilled from *A* to *B*. In the end, *B* is required to solve both tasks. The learning without forgetting (LWF) approach of Li and Hoiem (2018) is an example of a knowledge distillation technique (Parisi et al., 2019).

#### Rehearsal

Rehearsal approaches save raw data samples of previous tasks and incorporate them into the new task's training set. These samples can either be randomly or carefully chosen to save representatives of past tasks. The Incremental Classifier and Representation Learning (iCaRL) (Rebuffi et al., 2016) is an example rehearsal approach that keeps the most representative samples of previous tasks for future learning. This strategy allows weight strengthening for already learned memories.

#### Generative Replay

Compared to rehearsal approaches, generative replay (or pseudo-rehearsal) algorithms learn to artificially generate data samples for past tasks instead of saving raw data of previously seen tasks. Generative models learn the distribution of data previously encountered to replay past experiences when learning on new data. These models are often generative adversarial networks or auto-encoders (Lesort et al., 2020). An example is the generative replay approach (GR) proposed by Shin et al. (2017).

#### Hybrid

According to Lesort et al. (2020), most LL approaches rely on more than one of the four mentioned strategies, often leading to better solutions. One example is the previously mentioned iCaRL approach of Rebuffi et al. (2016). Additionally to the usage of raw data of past tasks, iCaRL uses knowledge distillation. Instead of transferring information between different neural networks, this knowledge is transferred within a single model between different time steps, similarly to the LWF (Li and Hoiem, 2018) approach. The Learning a Unified Classifier Incrementally via Rebalancing (LUCIR) (Hou et al., 2019) is similar to iCaRL. However, they introduce three new components to mitigate catastrophic forgetting caused by the imbalance between new and old data. This imbalance is also tackled by the Bias Correction (BiC) approach (Wu et al., 2019). The authors show evidence that the last fully connected layer of a CNN has a strong bias toward new classes, which they corrected by utilizing a bias correction layer after the last network layer. A further rehearsal and distillation regularization approach is the Pooled Outputs Distillation for Small-Task Incremental Learning (PODNet) (Douillard et al., 2020). The authors introduce a novel distillation-loss to ensure a balance between reducing forgetting and learning new tasks for long-term incremental learning, as well as a multi-mode similarity classifier that is more robust to data distribution shifts. The Dynamic Expandable Network (DEN) of Yoon et al. (2018) is a regularization and an architectural approach. It identifies neurons in a deep neural network relevant for a new task and selectively retrains them. However, if this selective retraining fails to achieve a desired loss, the network is expanded with additional neurons while unnecessary units are eliminated. Catastrophic forgetting is mitigated by duplicating neurons that drifted too much from their original values. Hou et al. (2018) proposed the Adaptation by Distillation approach, which combines knowledge distillation from an intermediate *Expert CNN* to learn new tasks with caching of small data subsets of previous tasks to preserve old knowledge during training a CNN. Therefore, it is a regularization and a rehearsal approach. The Dynamic Generative Memory (DGM), a combination of generative replay and dynamic architecture model, is presented by Ostapenko et al. (2019). A generative model is trained incrementally to learn task distributions over time. Samples of the current task and synthesized samples of all previous tasks are used to train a task solver. Additionally, the generative model can be expanded to ensure constant expressive power and sufficient capacity. The Riemannian Walk (RWalk) approach of Chaudhry et al. (2018) is a rehearsal and penalty computing regularization technique based on Kullback–Leibler-divergence (between output distributions) for parameter importance score computation. Representative samples of previous tasks are stored for replay to improve performance. A combination of regularization and implicit dynamic architecture approach, called Continual Learning with Adaptive Weights (CLAW), is presented by Adel et al. (2020). The architecture adaptation is data-driven by learning which neurons need to be trained and what is the maximum adaptation applied to these neurons using variational inference. The Incremental Learning With Dual Memory (IL2M) (Belouadah and Popescu, 2019) consists of two parts: (1) a deep learning model that is incrementally trained on new samples and a constant number of previous representative samples, and (2) an additional memory that stores previous task statistics which are periodically used to rectify the network. The Growing Dual Memory (GDM) approach of Parisi et al. (2018) is an architectural and a generative replay approach. In Subsection 2.2, it is described in more detail.

### 2.2. Growing Dual-Memory

The Growing Dual-Memory consists of two interconnected recurrent Growing When Required (GWR) (Marsland et al., 2002) models called *growing episodic memory* (G-EM) and *growing semantic memory* (G-SM), where both are extended versions of the *Gamma-GWR* model proposed by Parisi et al. (2017). Depending on the similarity between the input and the nearest neighbor in the network, i.e., the best-matching unit (BMU), new neurons are created or existing neurons trained. This similarity (or activation) is computed using the exponential function of the negative Euclidean distance. If the similarity is too low, compared to a fixed predefined activation threshold, a new neuron is created. Otherwise, the BMU and its neighbors are trained toward the input. Additional context vectors are used for each neuron. These context vectors are integrated into the similarity computation, allowing the utilization of past network activations to learn the input's temporal structure (Parisi et al., 2018). In both G-EM and G-SM, edges that exceed a predefined maximum age threshold are considered to be “too old” and are, therefore, removed. Isolated nodes are deleted. In the G-EM layer, temporal connections between neurons are introduced where consecutively activated units show a stronger temporal link. Given a neuron *s*(*i* − 1), the temporal connections allow the determination of the most probable next neuron *s*(*i*) of a prototype trajectory. These trajectories are replayed to G-EM and G-SM in the absence of external input. The weights of the BMU inside the G-EM and its temporal information are used to train the G-SM layer. In G-SM, Parisi et al. (2018) equipped the Gamma-GWR network with two additional constraints. It will only create new nodes if it cannot predict the current input correctly, and it will only update nodes if the input label can be adequately predicted. Both constraints are realized by maintaining an associative matrix *H*(*j, l*) for label predictions. It stores a histogram of input labels for each node *j*. The prediction of the GDM network is the label the BMU most frequently observed in the past. Additionally, Parisi et al. (2018) used a pre-trained and fine-tuned VGG-16 CNN (Simonyan and Zisserman, 2014) as a feature extractor to create 256-dimensional feature vectors for each input image. Transfer learning was performed using the CORe50 dataset (Subsection 2.4). These feature vectors are then used as the input for the G-EM layer.

### 2.3. Self-Organizing Incremental Neural Network+

The Self-organizing Incremental Neural Network+ approach of Wiwatcharakoses and Berrar (2019) has similarities to the Gamma-GWR architecture. Nevertheless, it differs fundamentally in how the deletion and creation of nodes or edges take place. The SOINN+ approach considers the deletion of edges and nodes as an intrinsic part of the learning itself. It deletes edges and nodes only if they are not relevant enough for the learning task (Wiwatcharakoses and Berrar, 2019). The key aspects of the SOINN+ approach are the *lifetime* of an edge as well as the *trustworthiness, idle time*, and *(un-)utility* of a node. *Trustworthiness* is used to determine whether to link two nodes with an edge or not. Particularly, the more trust there is in these nodes, the more likely they are connected. The more often a node was chosen as the BMU, the higher is the trust in it. This technique tries to avoid connecting nodes that represent noise. The *lifetime/age* of already deleted edges and the lifetime of all edges reachable from the BMU are used to calculate an edge pruning threshold. If the lifetime of an edge exceeds this threshold, it is removed. This technique enables the deletion of edges that connect nodes of different clusters to isolate these. The *unutility* is the ratio between the idle time and the winning time of a node. The former represents the number of iterations passed since the node was a BMU, and the latter the number of times it was chosen as the BMU. A node pruning threshold is calculated using the unutilities of deleted and existing nodes. If the unutility of a node exceeds this threshold, it is a candidate for deletion.

### 2.4. Benchmark Datasets

Most LL benchmark datasets are adopted from other fields not explicitly created for LL (Lesort et al., 2020). The samples of these datasets are split, artificially modified (e.g., rotation or permutation), or concatenated to create a sequence of tasks (Lesort et al., 2020). Examples are the permuted MNIST used by Kirkpatrick et al. (2016), the rotated MNIST (Lopez-Paz and Ranzato, 2017), or the incremental CIFAR100 (Rebuffi et al., 2016). Real-world datasets suitable for LL are the iCubWorld28 (Pasquale et al., 2015) or the iCubWorld Transformations (Pasquale et al., 2016; Pasquale et al., 2017). In these datasets, a human operator manipulates different objects in front of the iCub (Metta et al., 2008) robot's cameras. A tracking routine is used to move the robot's gaze toward the object and extract a bounding box around it. Lomonaco and Maltoni (2017) proposed the CORe50, a dataset specially designed for continuous object recognition. Fifty different objects belonging to 10 object categories are recorded. Each category consists of five instances. In contrast to the iCubWorld datasets, each object is recorded in front of 11 different real-world environments, both outdoor and indoor. In each video (15 s, 20 fps), a human hand moves and rotates the object smoothly in front of a camera. A motion-based tracker is used to create images with a resolution of 128 × 128 pixels by cutting out a bounding box around the object. However, both dataset acquisition processes are not controlled enough for perfect reproducibility. Besides, the ability to change individual aspects in the scenario (e.g., the background) without changing others in any way is not given. Nevertheless, this is desirable to analyze the behavior of LL models on particular environmental changes. The Toys-200 dataset of Stojanov et al. (2019) is recorded in a 3D virtual environment and consists of 200 toy-like objects. These are translated and scaled in front of a moving camera. The background contains further objects of the dataset distributed over a floor. In contrast to the CORe50 and the iCubWorld datasets, it is highly reproducible. However, it contains no operator (human or robot) who manipulates the objects, which would be the case in a long-term HRI. Such manipulation can lead to occlusion, making the task more difficult. Furthermore, except for the floor scenario, no real-world background images are considered.

## 3. Methodology

### 3.1. Associative SOINN+

**Figure 1:**
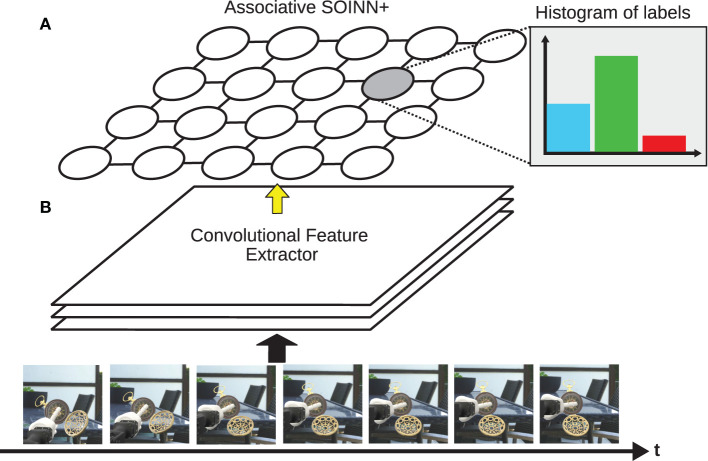
This figure shows an illustration of the A-SOINN+ **(A)** and the feature extractor **(B)**. Over time, different frames become available. These are fed into the feature extractor, illustrated by the thick, black arrow. The feature extractor creates feature vectors representing the image frames. These feature vectors are fed into the A-SOINN+ model afterward, illustrated by the thin, yellow arrow. The proposed model stores a histogram of input labels for each node to facilitate classification, shown for one node in the top right corner. This figure is inspired by the GDM illustration of Parisi et al. (2018). The 3D object is a derivative of “Antique Pocket Watch” by “michael_grodkowski” licensed under CC BY 4.0.

The associative SOINN+ (A-SOINN+) extends the SOINN+ of Wiwatcharakoses and Berrar (2019) in two points. Both are adopted from the GDM approach. (1) An associative matrix *H*(*j, l*) is maintained, which stores how often a neuron observed a particular input label. This point gives this extension its name and enables classification, which was previously not possible in the SOINN+. (2) Top-down cues to regulate the network's structural plasticity are introduced in the form of additional constraints for node creation and weight adaptation. Additionally, inspired by Parisi et al. (2018), a pre-trained and fine-tuned VGG-16 (Simonyan and Zisserman, 2014) CNN architecture is used as a feature extractor to extract the most relevant features out of images and reduce the dimensionality. The A-SOINN+ is an architectural LL approach as it adapts its shape in response to novel input. In contrast to GDM, it does not perform memory replay and is, therefore, no hybrid approach. The architecture of the A-SOINN+ and the feature extractor are illustrated in Figure 1.

Furthermore, the (associative) SOINN+ algorithm^1^ is shown in algorithms 1–6. The adaptations made in this work have the prefix: **(A-SOINN+)**.

#### Main Routine

The network is initialized with three random, unconnected neurons with a winning time of one and an idle time of zero (Algorithm 1, steps 1–4). The associative matrix is initially empty (Algorithm 1, Step 5). As soon as a new training sample *x*(*t*) becomes available, the BMU *b* and sBMU *s* are computed using the Euclidean distance (Algorithm 1, step 7). Furthermore, the associative matrix entries of the BMU *b* are updated, such that *H*(*b, l*(*t*)) is increased by a predefined δ^+^ and *H*(*b, k*) decreased by δ^−^ for all *k* ≠ *l*(*t*) (Algorithm 1, steps 8 and 9) (Parisi et al., 2017). The similarity thresholds of *b* and *s* are calculated afterward (Algorithm 2) using:

(1) $$\begin{array}{l} \tau \left(i\right)=\{\begin{array}{ll} \max_{j\in N_{i}}\|w_{i}-w_{j}\|, & \text{if }N_{i}\neq \emptyset \\ \min_{j\in A\backslash \left\{i\right\}}\|w_{i}-w_{j}\|, & \text{otherwise} \end{array}\text{,} \end{array}$$

for *i* ∈ [*b, s*]. The weight vector is denoted by *w*_*i*_ and the neighbors of *i* by *N*_*i*_. Hence, the similarity threshold of a node *i* is the maximum distance from *i* to all of its neighbors. If *i* has no neighbors, τ(*i*) is the distance between *i* and its closest node. The prediction (Algorithm 1, Step 11) of the network is the label the BMU observed most frequently, computed with:

(2) $$\begin{array}{l} \xi_{b}=\underset{l\in L}{arg max}H\left(b,l\right). \end{array}$$

Suppose the Euclidean distance between the input *x*(*t*) and *b* or between *x*(*t*) and *s* is larger than the corresponding similarity threshold, and simultaneously the prediction ξ_*b*_ of *b* is unequal to the input label *l* (i.e., creation condition is true). In that case, a new node is created (Algorithm 1, Step 15). The input *x*(*t*) is used as the weight vector of the new node. Winning time and idle time are initiated with one and zero, respectively. If, on the other hand, no neuron is created, three subroutines are executed, namely node merging, node linking, and edge deletion (Algorithm 1, steps 16–19), but only if the input label is unequal to the prediction (i.e., adaptation condition is true). The node deletion subroutine is performed at the end (Algorithm 1, Step 21).

**Algorithm 1:** (Associative) Self-Organizing Incremental Neural Network +

1: *A* ← Set of neurons with random weight vectors.
2: *WT*(*i*) ← 1, ∀*i* ∈ [1, 2, 3] // Set the winning time to one.
3: *IT*(*i*) ← 0, ∀*i* ∈ [1, 2, 3] // Set the idle time to zero.
4: *E* ← ∅ // Initialize an empty set of connections.
5: **(A-SOINN+)**: *H*(*i*, ·) ← ∅, ∀*i* ∈ [1, 2, 3] // Initialize an associative matrix with no labels
6: **for** each input *x*(*t*) with label *l*(*t*) **do**
7: $b\leftarrow {\text{arg min}}_{j\in A}{\left\|x\left(t\right)-w_{j}\right\|}^{2}$, // Find the BMU $s\leftarrow {\text{arg min}}_{j\in A\backslash \left\{b\right\}}{\left\|x\left(t\right)-w_{j}\right\|}^{2}$ // Find the (s)BMU.
8: **(A-SOINN+)**: Δ*H*(*b, l*(*t*)) = δ^+^, Δ*H*(*b, k*) = − δ^−^, ∀*k* ∈ *L*\{*l*(*t*)} // Update associative matrix
9: *N*_*b*_, *N*_*s*_ ← neighbors of *b*, *s*
10: τ(*b*), τ(*s*) ← Calculation of similarity thresholds (Algorithm 2)
11: **(A-SOINN+)**: ξ_*b*_ ← arg max_*l* ∈ *L*_*H*(*b, l*) // Get prediction (Equation 2).
12: **(A-SOINN+)**: creation_condition ← ξ_*b*_ ≠ *l*(*t*)
13: **(A-SOINN+)**: adaptation_condition ← ξ_*b*_ = *l*(*t*)
14: **if** (||*x*(*t*) − *w*_*b*_|| ≥ τ(*b*) **or** ||*x*(*t*) − *w*_*s*_|| ≥ τ(*s*)) **and** creation_condition **then**
15: *w*_*r*_ ← *x*(*t*), *A* ← *A* ∪ {*r*}, *WT*(*r*) ← 1, *IT*(*r*) ← 0 // Create a new node.
16: **else if** adaptation_condition **then**
17: Node merging (Algorithm 3)
18: Node linking (Algorithm 4)
19: Edge deletion (Algorithm 5)
20: **end if**
21: Node deletion (Algorithm 6)
22: **end for**

**Algorithm 2:** (Associative) SOINN+ Calculation of similarity thresholds

1: **for** *i* in [*b, s*] **do**
2: **if** *N*_*i*_ ≠ ∅ **then**
3: $\tau \left(i\right)\leftarrow \underset{j\in N_{i}}{\max}\|w_{i}-w_{j}\|$ // (Equation 1)
4: **else**
5: $\tau \left(i\right)\leftarrow \underset{j\in A\backslash \left\{i\right\}}{\min}\|w_{i}-w_{j}\|$ // (Equation 1)
6: **end if**
7: **end for**

#### Node Merging

In the node merging subroutine (Algorithm 3), the weights of *b* and its neighbors *i* ∈ *N*_*b*_ are adapted toward the input with:

(3) $$\begin{array}{l} \Delta w_{b}=\epsilon_{b}\cdot \frac{x\left(t\right)-w_{b}}{WT\left(b\right)}\text{,} \end{array}$$

(4) $$\begin{array}{l} \Delta w_{i}=\epsilon_{n}\cdot \frac{x\left(t\right)-w_{i}}{WT\left(i\right)}\text{.} \end{array}$$

While Wiwatcharakoses and Berrar (2019) present a pull factor η to regulate the neighboring neurons' inertia, we use the learning rate ϵ_*n*_ to resemble the GDM approach better. It can be considered as the inverse pull factor $\epsilon_{n}=\frac{1}{\eta }$. A further learning rate ϵ_*b*_ is introduced to facilitate a more controlled weight adaptation of the BMU. The amount of learning is also modulated by the winning time to avoid strong adaptations of well-trained units.

**Algorithm 3:** (Associative) SOINN+ Node Merging

1: *WT*(*b*) ← *WT*(*b*) + 1
2: **(A-SOINN+)**: $w_{b}\leftarrow w_{b}+\epsilon_{b}\cdot \frac{x\left(t\right)-w_{b}}{WT\left(b\right)}$ // Update BMU's weight vector (Equation 3).
3: **for** *i* in *N*_*b*_ **do**
4: $w_{i}\leftarrow w_{i}+\epsilon_{n}\cdot \frac{x\left(t\right)-w_{i}}{WT\left(i\right)}$ // Update the neighbors of b (Equation 4).
5: **end for**
6: *IT*(*b*) ← 0 // Set the idle time of the BMU to zero.

#### Node Linking

The node linking subroutine determines whether the BMU *b* and the sBMU *s* are connected (Algorithm 4). Here, no modifications are made compared to the original approach. A connection between *b* and *s* is created if at least one of three conditions (Algorithm 4, Step 9) is true. The first is that the number of edges in the network is lower than three to ensure edge creation in the initial training steps. The second and third conditions depend on the trustworthiness *T*(*i*) of a node *i*, which is defined as:

(5) $$\begin{array}{l} T\left(a\right)=\frac{WT\left(i\right)-1}{max\left(WT\right)-1}, \end{array}$$

with the maximum winning time of all nodes *max*(*WT*). Furthermore, the values $\bar{\tau_{b}}$, $\bar{\tau_{s}}$, σ_*b*_, and σ_*s*_ are required. Let τ_*b*_ be the set of similarity thresholds of each connected BMU (i.e., having at least one edge) ever encountered. $\bar{\tau_{b}}$ is the arithmetic mean of τ_*b*_ and σ_*b*_ the corresponding standard deviation. $\bar{\tau_{s}}$ and σ_*s*_ are analogously defined for the sBMUs. The two additional conditions for node linking are defined as:

(6) $$\begin{array}{l} \tau \left(b\right)\cdot \left(1-T\left(b\right)\right)<\bar{\tau_{b}}+2\cdot \sigma_{b}\text{,} \end{array}$$

(7) $$\begin{array}{l} \tau \left(s\right)\cdot \left(1-T\left(s\right)\right)<\bar{\tau_{s}}+2\cdot \sigma_{s}\text{.} \end{array}$$

Hence, the more trust there is in *b* or *s*, the more likely they are linked. Using this conditional connection technique tries to connect nodes that do not represent noise (Wiwatcharakoses and Berrar, 2019).

**Algorithm 4:** (Associative) SOINN+ Node linking

1: **for** *i* in *A* **do**
2: *max*(*WT*) ← Maximum winning time of all nodes
3: $T\left(i\right)\leftarrow \frac{WT\left(i\right)-1}{max\left(WT\right)-1}$ // Update trustworthiness of each node (Equation 5).
4: **end for**
5: $\bar{\tau_{b}}$, σ_*b*_ ← mean and standard deviation of the similarity thresholds of all BMUs with an edge to sBMU
6: $\bar{\tau_{s}}$, σ_*s*_ ← mean and standard deviation of the similarity thresholds of all sBMUs with an edge to BMU
7: condition1 $\leftarrow \tau \left(b\right)\cdot \left(1-T\left(b\right)\right)<\bar{\tau_{b}}+2\cdot \sigma_{b}$ // (Equation 6)
8: condition2 $\leftarrow \tau \left(s\right)\cdot \left(1-T\left(s\right)\right)<\bar{\tau_{s}}+2\cdot \sigma_{s}$ // (Equation 7)
9: **if** (|*E*| < 3) **or** condition1 **or** condition2 **then**
10: **if** (*b, s*) ∉ *E* **then**
11: *E* ← *E*∪{(*b, s*)} // Create an edge between the BMU and the sBMU.
12: Update $\bar{\tau_{b}}$, $\bar{\tau_{s}}$, σ_*b*_, σ_*s*_
13: **end if**
14: **end if**
15: **if** (*b, s*) ∈ *E* **then**
16: *LT*(*b, s*) ← 0 // Set the lifetime of the BMU, sBMU edge to zero.
17: **end if**
18: **for** all *i* in *N*_*b*_ **do**
19: *LT*(*b, i*) ← *LT*(*b, i*) + 1 // Update the lifetime of all neighbors of the BMU.
20: **end for**

#### Edge Deletion

The lifetime (or age) *LT*(*i, j*) of an edge *e* = (*i, j*) plays a crucial role in the edge deletion subroutine (Algorithm 5). If *e* is connected to the BMU *b* and the sBMU *s*, its lifetime is reset to one (Algorithm 4, Step 16). If it is connected to *b* but not to *s*, it is increased by one (Algorithm 4, Step 19). First, the lifetimes of all edges that can be reached from *b* are computed ($L_{b}$) (Algorithm 5, Step 1). Edges with an exceptionally high lifetime (i.e., outliers) are detected using the threshold:

(8) $$\begin{array}{l} \omega_{edge}=L_{0.75}+2\cdot IQR\left(L_{b}\right). \end{array}$$

$L_{0.75}$ represents the 75^*th*^ percentile of the lifetimes in $L_{b}$ and $IQR\left(L_{b}\right)=L_{0.75}-L_{0.25}$ the interquartile range (Upton and Cook, 1996). Additionally, the average lifetime of all deleted edges ${\bar{L}}_{del}$, as well as the number of deleted edges $\left|L_{del}\right|$, are considered, resulting in the final threshold:

(9) $$\begin{array}{l} \lambda_{edge}={\bar{L}}_{del}\cdot \frac{\left|L_{del}\right|}{\left|L_{del}|+|L_{b}\right|}+\omega_{edge}\cdot \left(1-\frac{\left|L_{del}\right|}{\left|L_{del}|+|L_{b}\right|}\right). \end{array}$$

Edges connected to *b* with a higher lifetime than λ_*edge*_ are deleted. This deletion technique removes edges that connect nodes of different clusters to isolate those (Wiwatcharakoses and Berrar, 2019).

**Algorithm 5:** (Associative) SOINN+ Edge deletion

1: $L_{b}\leftarrow$ set of lifetimes of edges through which the BMU can be reached
2: $L_{0.75}\leftarrow 75th$ percentile of elements in $L_{b}$
3: $\omega_{edge}\leftarrow L_{0.75}+2\cdot IQR\left(L_{b}\right)$ // (Equation 8)
4: $\lambda_{edge}\leftarrow {\bar{L}}_{del}\cdot \frac{\left|L_{del}\right|}{\left|L_{del}|+|L_{b}\right|}+\omega_{edge}\cdot \left(1-\frac{\left|L_{del}\right|}{\left|L_{del}|+|L_{b}\right|}\right)$ // (Equation 9)
5: **for** all *i* in *N*_*b*_ **do**
6: **if** *LT*(*b, i*) > λ_*edge*_ **then**
7: *E* ← *E*\{(*b, i*)} // Remove the edge.
8: **(A-SOINN+)**: Update $\left|L_{del}\right|$, $L_{del}^{sum}$, and ${\bar{L}}_{del}$
9: **end if**
10: **end for**

#### Node Deletion

The node deletion subroutine is executed independently of whether a node was added or merged (Algorithm 1, Step 21). The unutility of a node *i* is defined as:

(10) $$\begin{array}{l} U\left(i\right)=\frac{IT\left(i\right)}{WT\left(i\right)}. \end{array}$$

If a node was rarely chosen as the BMU, its unutility is high. On the other hand, it is low if the node was relatively often a BMU. Let U be the set of unutilities of not isolated nodes in the network. The threshold for the outlierness of an unutility is defined as:

(11) $$\begin{array}{l} \omega_{node}=median\left(U\right)+2\cdot sMAD\left(U\right), \end{array}$$

with the scaled median absolute deviation (sMAD) (Leys et al., 2013) of the elements in U. Wiwatcharakoses and Berrar (2019) do not explain why the sMAD is used here and the IQR in the edge deletion subroutine. We assume that they achieved a higher performance with this configuration. Let $\left|U_{del}\right|$ be the number of deleted nodes and ${\bar{U}}_{del}$ the average unutility of these deleted nodes. Furthermore, let $R_{noise}=\frac{\left|I\right|}{\left|A\right|}$ be the ratio between the unconnected nodes I and all nodes *A*. The threshold for node deletion is then defined as:

(12) $$\begin{array}{l} \lambda_{node}={\bar{U}}_{del}\cdot \frac{\left|U_{del}\right|}{\left|U_{del}|+|A\backslash I\right|}+\omega_{node}\cdot \left(1-\frac{\left|U_{del}\right|}{\left|U_{del}|+|A\backslash I\right|}\right)\cdot \left(1-R_{noise}\right). \end{array}$$

Three conditions are required to be fulfilled for a node *i* to be deleted: (1) at least one edge exists in the network, (2) the unutility of *i* is larger than λ_*node*_, and (3) *i* is unconnected.

#### Further Adaptations

For efficiency reasons, further adaptations to the original algorithm are performed. Instead of maintaining three sets of deleted parts of the network, i.e., the set of all deleted nodes *A*_*del*_, the set of all deleted unutilities $U_{del}$, and the set of lifetimes of deleted edges $L_{del}$, the A-SOINN+ approach only maintains the properties of those sets. Notably, by continuously updating the sizes $\left|U_{del}|=|A_{del}\right|$ and $\left|L_{del}\right|$, the sums $U_{del}^{sum}$ and $L_{del}^{sum}$, as well as the mean values ${\bar{U}}_{del}$ and ${\bar{L}}_{del}$ (Algorithm 5, Step 8 and Algorithm 6, Step 12). Thus, three sets are replaced by six values, reducing the memory requirements, especially for large numbers of deleted nodes and edges. We utilized context learning for the A-SOINN+ in the first place. However, preliminary tests showed no improvements if doing so. Hence, we do not consider context learning in the description of the algorithm.

**Algorithm 6:** (Associative) SOINN+ Node deletion

1: **for** *i* in *A* **do**
2: $U\left(i\right)\leftarrow \frac{IT\left(i\right)}{WT\left(i\right)}$ // Update unutilities of all nodes (Equation 10).
3: **end for**
4: U← Set of unutilities of all nodes with edges
5: I← Set of unconnected nodes
6: $\omega_{node}\leftarrow median\left(U\right)+2\cdot sMAD\left(U\right)$ // (Equation 11)
7: $R_{noise}\leftarrow \frac{\left|I\right|}{\left|A\right|}$
8: $\lambda_{node}\leftarrow {\bar{U}}_{del}\cdot \frac{\left|U_{del}\right|}{\left|U_{del}|+|A\backslash I\right|}+\omega_{node}\cdot \left(1-\frac{\left|U_{del}\right|}{\left|U_{del}|+|A\backslash I\right|}\right)\cdot \left(1-R_{noise}\right)$ // (Equation 12)
9: **for** *i* in *A* **do**
10: **if** |*E*| > 1 **and** *U*(*i*) > λ_*node*_ **and** *i* has no edges **then**
11: *A* ← *A*\{*i*} // Delete the node.
12: **(A-SOINN+)**: Update $\left|U_{del}\right|$, $U_{del}^{sum}$, and ${\bar{U}}_{del}$
13: **end if**
14: **end for**
15: **for** *i* in *A* **do**
16: *IT*(*i*) ← *IT*(*i*) + 1 // Increment the idle time of each node.
17: **end for**

### 3.2. v-NICO-World-LL, a New LL Dataset

In long-term HRI, robots are required to actively explore and learn about their environment while interacting with a human. Part of this is grasping items and viewing them from different angles (along with collecting other multi-modal data, like tactile information). We make a step toward this behavior with our proposed LL dataset, the “virtual NICO World for Lifelong Learning”(*v-NICO-World-LL*). It is inspired by the CORe50 of Lomonaco and Maltoni (2017) but exhibits three novel features currently not considered by other object recognition LL datasets:

1. The environments are grouped into different levels of complexity.
2. A robot is manipulating the objects.
3. The dataset is recorded in a nearly photorealistic controlled virtual environment.

**Figure 2:**
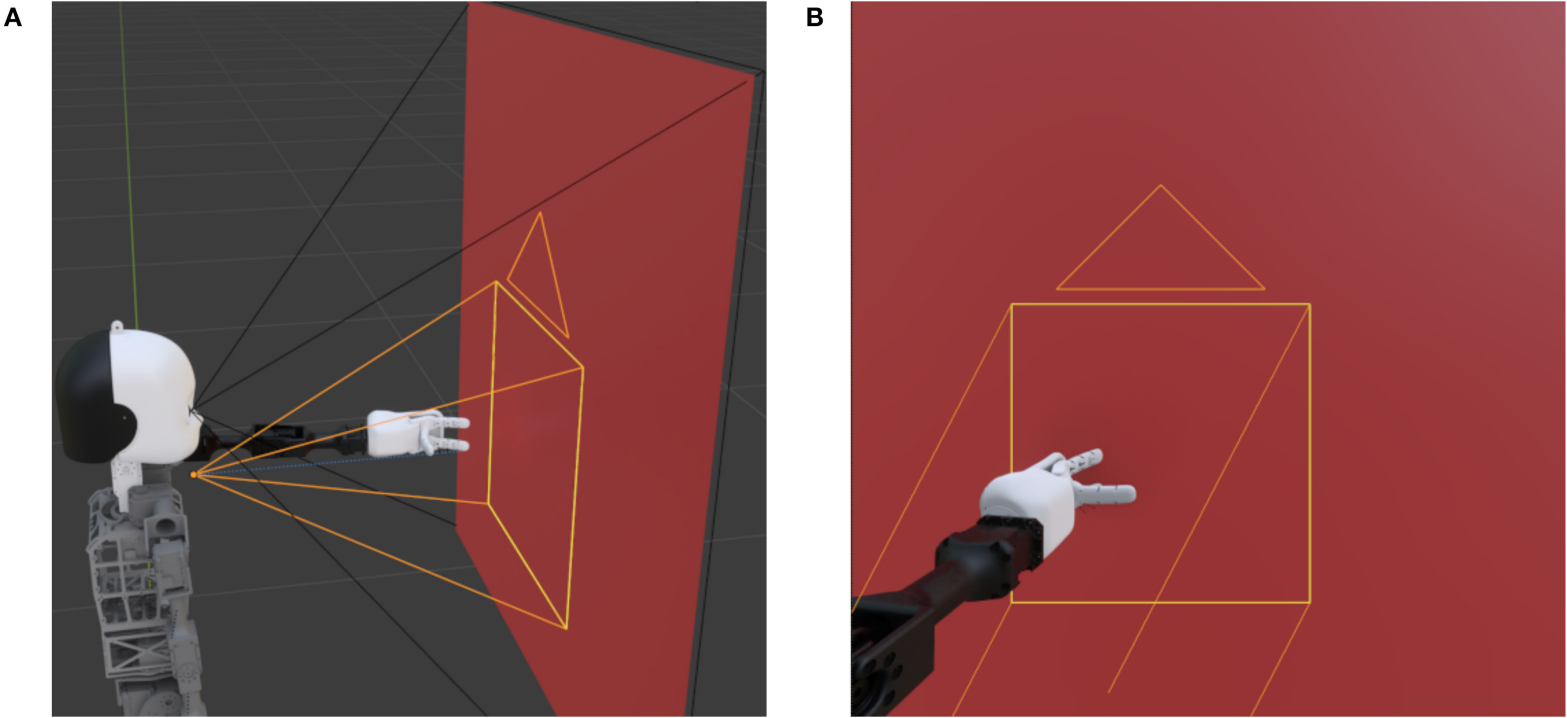
The Blender scenario from two different perspectives. **(A)** shows the robot, the plane, and the two fields of vision as black and orange lines. While the former lines represent the fixed main camera positioned at the right eye, the latter represent the moving camera focused on the robot's hand. **(B)** is showing the perspective of the main camera.

The proposed dataset is recorded in a controlled virtual environment in Blender (Blender Foundation, 2020) with a 3D model of the humanoid robot NICO (Kerzel et al., 2017; Kerzel et al., 2020) developed by the Knowledge Technology Group at the Universität Hamburg. Using a virtual environment facilitates a controlled and reproducible data acquisition process. In the CORe50 and the iCubWorld datasets, a human operator is holding the objects. However, in a long-term HRI scenario, a robot needs to recognize objects in its hand as well. We fill this gap by placing objects in the robot's left hand. This aspect leads to a different perspective on the object. Furthermore, the robot hand differs from a human hand due to its shape and color (e.g., it has only three fingers and is white-colored). This results in different occlusions and contrasts compared to a human hand. The fingers are positioned so that it looks as if the robot is holding the object. Figure 2A shows the general scenario. NICO starts with an outstretched arm in front of a background panel. The black lines demonstrate the field of vision of the main camera placed at the right eye and filling the entire background panel. The same scenario but from the main camera's perspective is shown in Figure 2B. Since only a fraction of the frames contains the object of interest, cropped images focusing on the object are created using a second camera (orange lines). This camera moves in two dimensions (i.e., left/right and up/down) to follow the robot's hand.

**Figure 3:**
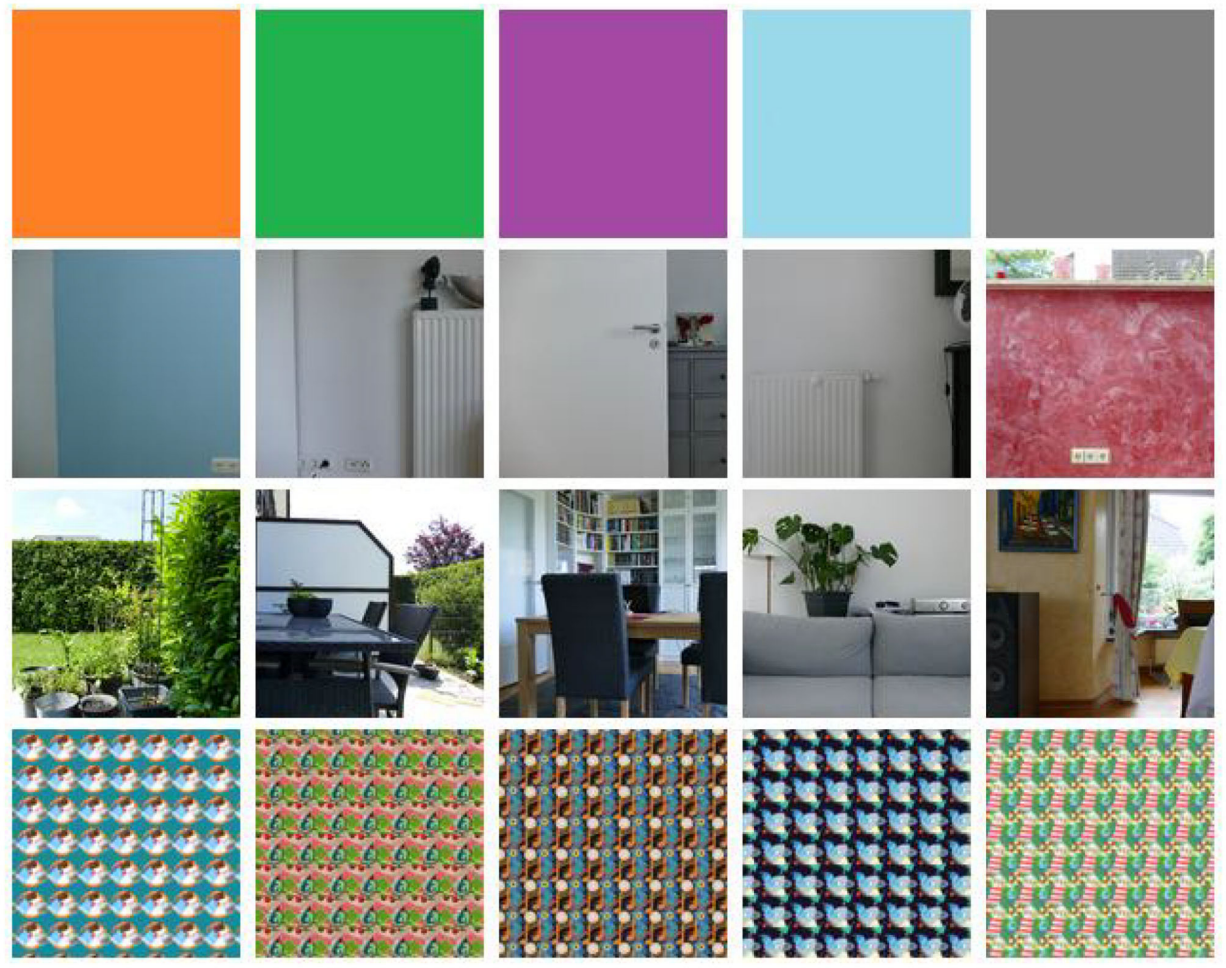
The 20 different backgrounds of the dataset. The images are split into four complexity groups (B1, B2, B3, and B4), each shown in one row with increasing complexity from top to bottom. The first row shows the plain colored background images of B1. B2 and B3 (second and third row, respectively) are showing real-world backgrounds. A camera captured the B2 and B3 examples from a height of 89 cm. B4 (fourth row) consists of artificial background images with different colors and drawn objects.

**Figure 4:**
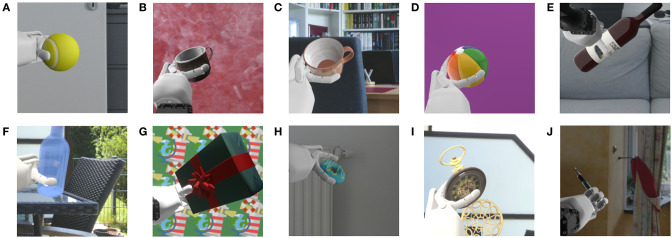
This figure presents 10 example images of the dataset captured from the moving camera's perspective. Ten objects are shown in front of different backgrounds with different lighting conditions. Additionally, NICO is holding the objects in different positions. **(A)** 3D model of “Tennis ball” by “tr3vinj”, **(B)** “Metal Cup” by “Warkarma”, **(C)** “Mug” by “Kristijan Zecevic”, **(D)** “Beach Ball” by “Tommyleenev”, **(E)** “3D Wine Bottle Model” by “Johana-PS”, **(F)** “Glass Bottle” by “Aullwen”, **(G)** “Gift - 3December” by “zhixson”, **(H)** Derivative of “Coffee and donuts” by “Ivan Dnistrian”, **(I)** Derivative of “Antique Pocket Watch” by “michael_grodkowski”, and **(J)** Derivative of “Fountain Pen” by “Etherlyte”. All 10 models are used under CC BY 4.0 and also licensed under CC BY 4.0.

The robot's arm is moving smoothly in a 10-s animation by manipulating an elbow, a wrist, and a palm joint, as well as three shoulder joints. RGB images are rendered with 24 fps from the perspective of both cameras. The image resolutions are 512 × 512 pixels and 256 × 256 pixels for the main and moving camera, respectively. The dataset consists of 100 objects belonging to 10 categories: ball, book, bottle, cup, doughnut, glasses, pen, pocket watch, present, and vase. These objects are chosen such that they could appear in a long-term HRI scenario. The 10 instances of a category *C* are denoted with *I*_0_ to *I*_9_ throughout this work. All objects are downloaded from the four websites: https://www.turbosquid.com, https://sketchfab.com, https://free3d.com, and https://www.cgtrader.com in the period from April 26, 2020, to June 6, 2020. They are slightly modified in Blender to fit into the scenario. The objects are manipulated in front of 20 different backgrounds, divided into four background complexities, each with five different background instances. These different background complexities allow the controlled evaluation of how LL models respond to different degrees of environmental conditions. Figure 3 shows all backgrounds. Each row represents a background complexity, and each column an instance of this complexity. *Background complexity* 1 (B1), shown in the first row of Figure 3, is composed of five plain colored images. Real-world background images are used in B2 and B3 (second and third row, respectively) to simulate different environments where long-term HRI can occur. While complexity B2 consists of images with low variability (i.e., simple walls with little content), B3 contains more complex indoor and outdoor scenes with different visible objects, like furniture or plants. The real robot's total height is 101 cm (Kerzel et al., 2017), such that the cameras of NICO are approximately 89 cm above the ground. Hence, each background image of B2 and B3 is captured from a height of 89 cm to create more realistic recordings. B4 (fourth row) comprises artificial images with a cluttered dynamic structure where drawn objects move in the background. Each instance of B2, B3, and B4 has a different lighting condition in the virtual environment. In B2 and B3, the real-world lighting conditions are tried to be reproduced. By grouping the background images into different complexity levels, the influence of different degrees of environmental conditions on a model can be investigated, making it the key feature of the v-NICO-World-LL dataset. Ten example images of the dataset captured from the moving camera's perspective are shown in Figure 4. It illustrates the dataset's diversity regarding the background, lighting condition, and NICO's hand position. In total, the dataset consists of two times 2,000 videos (cropped and non-cropped), each having 240 frames, resulting in two times 480,000 RGB images.^2^

## 4. Experiments and Results

### 4.1. Experimental Setup

Five different experiments, organized into two parts, are conducted. Each experiment considers different data samples. The background complexities B1, B2, B3, and B4, are utilized in the first four experiments. In the last experiment, the CORe50 is used to determine the robustness of the A-SOINN+ against a different and well-known dataset. In each experiment, three GDM models with different maximal ages are compared to two versions of the A-SOINN+. The considered maximal ages are 6,000, 12,000, and 28,000. These models are referred to as *GDM 6,000, GDM 12,000*, and *GDM 28,000*, respectively. Empirical tests have shown that these maximal age values exhibit a good trade-off between accuracy and neuron number. Additionally, we investigate the performance of the A-SOINN+ with and without the node creation constraint to examine this constraint's influence on neuron number and accuracy. The A-SOINN+ without creation constraint is referred to as (ncc)A-SOINN+. We focus on analyzing this constraint as it is directly linked to the two criteria, which we apply to evaluate the different approaches: the task performance and the number of created nodes. In each experiment, the models are required to learn 10 different tasks. A task *t*_*i*_ of time step *i* refers to learning to classify a particular category of the v-NICO-World-LL or the CORe50 dataset. We compare the A-SOINN+ only to the GDM due to two reasons. First, the GDM shows state-of-the-art LL performance compared to other LL approaches (Parisi et al., 2018). Second, both A-SOINN+ and GDM show a strong architectural resemblance as they are based on the GWR (Marsland et al., 2002) approach. In future work, further comparisons can be examined.

#### v-NICO-World-LL Experiments

For the first four experiments, each frame is transformed into a feature vector using a VGG-16 (Simonyan and Zisserman, 2014) feature extractor. It is pre-trained on the ImageNet (Russakovsky et al., 2015) dataset and fine-tuned using the v-NICO-World-LL. The original VGG-16 is adapted to have two 2048-dimensional and one 128-dimensional fully connected hidden layers after the last pooling layer. The output layer is 10-dimensional (one for each category). Each frame is transformed using the third hidden layer's 128-dimensional output. In contrast to Parisi et al. (2018), we use 128-dimensional vectors instead of 256-dimensional to reduce the training time. The first three instances (*I*_0_-*I*_2_) of each category are used to train the four mentioned fully connected layers. Instances *I*_3_ to *I*_7_ are used to validate the model and *I*_8_ and *I*_9_ to test it. Five runs are performed with a particular hyperparameter assignment, resulting in a test accuracy of 84.2 ± 0.32% averaged across these runs. No hyperparameter optimization is done here since it is not essential to find an optimal accuracy in this work. Instead, sufficiently high accuracy results for reliable feature vector creation are required. A video is represented by a *feature vector sequence* (FVS) of the corresponding frames. Only each second frame of a video is considered for training to reduce the training time further.

At each iteration *i*, a task-dependent training set $Tr_{i}^{\prime }$ is used to train the models. It contains the FVS of the training instances *I*_*i*,3_ to *I*_*i*,7_ of the category *C*_*i*_. The first three instances of each category (*I*_0_-*I*_2_) are not considered in the LL experiments since the feature extractor is trained on these. This strategy avoids that the feature extractor learns “future” objects on which the LL models are later trained. This contrasts with the work of Parisi et al. (2018), where the feature extractor was trained on all object instances. After each learned task, task-dependent test sets are used to measure the performance of each model. Single-head evaluation (Chaudhry et al., 2018) is performed, i.e., a task-dependent test set *Te*_*i*_ contains the instances *I*_*i*,8_ and *I*_*i*,9_, as well as *I*_*j*,8_ and *I*_*j*,9_ of all previous tasks *t*_*j*_, with *j* < *i*. Hence, the models are required to predict the category of the current task and the previous tasks. Additionally, the networks are not only tested on the test set *Te*_*i*_ after learning *t*_*i*_ but also all previous *Te*_*j*_, with *j* < *i*. Three metrics are investigated: the average accuracy (Definition 1), the average forgetting (Definition 2), and the number of neurons. For the GDM models, the units of the G-EM and G-SM layers are summed up.

**Definition 1**. (Average Accuracy Chaudhry et al., 2018)

Let *a*_*k,j*_ ∈ [0, 1] be the accuracy evaluated on the test set *Te*_*j*_ of task *t*_*j*_ with *j* ≤ *k* after training the model incrementally from *t*_1_ to *t*_*k*_. The average accuracy at task *t*_*k*_ is:

$$\begin{array}{l} A_{k}=\frac{1}{k}\overset{k}{\underset{j=1}{\sum }}a_{k,j}. \end{array}$$

**Definition 2**. (Average Forgetting Chaudhry et al., 2018) Forgetting $f_{j}^{k}\in \left[-1,1\right]$ for a task *t*_*j*_, after the model learned from task *t*_1_ to *t*_*k*_, is the difference between the maximum knowledge gained about the task in the past and the knowledge the model currently has about it: $f_{j}^{k}=\underset{l\in \left\{1,\cdots \;,k-1\right\}}{\max}a_{l,j}-a_{k,j},\forall j<k.$ The average forgetting at task *t*_*k*_ is, therefore:

$$\begin{array}{l} F_{k}=\frac{1}{k-1}\overset{k-1}{\underset{j=1}{\sum }}f_{j}^{k}. \end{array}$$

Hence, *A*_*k*_ represents the performance of a model on the current task *t*_*k*_ and all previous tasks. *F*_*k*_ represents how much a model forgot about all previous tasks given the current time step *k*. The order of the tasks can affect the final results (Lomonaco and Maltoni, 2017; Parisi et al., 2018). Hence, each experiment is repeated four times, and the results are averaged. Each repetition has a different, randomly generated, category order:

- R1: ball, bottle, cup, doughnut, glasses, pen, pocket watch, present, vase, book
- R2: ball, book, bottle, cup, doughnut, glasses, pen, pocket watch, present, vase
- R3: doughnut, ball, pocket watch, glasses, pen, book, bottle, present, vase, cup
- R4: present, cup, pocket watch, vase, doughnut, pen, glasses, book, ball, bottle

The GDM, and the A-SOINN+ approach, have hyperparameters required to be tuned to preserve reliable models. Therefore, a grid search is performed for each model. Since each experiment requires 20 models (three GDM and two A-SOINN+ for each repetition), 20 grid searches are executed for a single experiment. The considered hyperparameters and assignments of the GDM and A-SOINN+ are shown in Tables 1, 2, respectively. Memory replay is used for each GDM model since Parisi et al. (2018) already showed that it improves the performance of the GDM approach. Preliminary tests confirmed this for the proposed dataset. According to Parisi et al. (2018), *K* values larger than two do not improve the GDM model's performance. Therefore, only two assignments are considered.

**Table 1:** This table shows the considered GDM hyperparameter assignments for the grid searches of the first four experiments.

**Parameter**	**Description**	**Assignments**
*K*	No. of context vectors	**2**, 4
[ϵ_*b*_, ϵ_*n*_]	Learning rates	[0.1, 0.001], **[0.3, 0.003]**, [0.5, 0.005]
$\left[a_{th}^{E},a_{th}^{S}\right]$	Activation thresholds	**[0.1, 0.01]**, [0.3, 0.03], [0.5, 0.05]

*K denotes the number of context vectors. ϵ_b_ and ϵ_n_ represent the learning rates of the BMU and its neighbors, respectively. The activation thresholds of the G-EM and the G-SM layers are denoted with $a_{th}^{E}$ and $a_{th}^{S}$, respectively. The best results averaged across all grid search iterations are shown in bold letters*.

**Table 2:** This table shows the considered A-SOINN+ hyperparameter assignments for the grid searches of the first four experiments.

**Parameter**	**Description**	**Assignments**
*K*	No. of context vectors	**2**, 4
[ϵ_*b*_, ϵ_*n*_]	Learning rates	[0.3, 0.003], [0.5, 0.005], [1, 0.001], [2, 0.001], **[3, 0.002]**

*K denotes the number of context vectors. ϵ_b_ and ϵ_n_ represent the learning rates of the BMU and its neighbors, respectively. The best results averaged across all grid search iterations are shown in bold letters*.

Both tables show the best assignments averaged across all grid search iterations in bold letters. We considered context learning for the A-SOINN+ in the first place. Therefore, we utilize the number *K* of context vectors in our grid searches. However, as preliminary tests showed, no improvements were observable if including context learning into the A-SOINN+. This grid search confirms this assumption since both assignments show similar average accuracy values (*K* = 2: 83.9%, *K* = 4: 83.7%). The best models of each grid search are used for a more detailed analysis by investigating the three mentioned metrics.

#### CORe50 Experiment

In the last experiment, we aim to maintain consistency with (Parisi et al., 2018), resulting in several differences compared to the previous four experiments. A new feature extractor is fine-tuned on the CORe50 to predict the 50 object instances of the dataset. The last hidden layer is 256-dimensional, resulting in 256-dimensional feature vectors. The CORe50 contains 11 backgrounds/sessions. Samples from sessions #3, #7, and #10 are used for testing the feature extractor and the remaining for training. After five runs with one particular parametrization, an average accuracy of 76.47% ± 0.21% is achieved. The same train/test split is used for LL afterward. Therefore, the task-dependent training set of a particular iteration contains all five instances of one object category from all sessions except #3, #7, and #10. Again four different repetitions are performed to reduce the category order influence. The randomly generated category orders are:

- R1: plug adapter, glasses, light bulb, scissors, cup, remote control, marker, can, mobile phone, ball
- R2: scissors, cup, plug adapter, light bulb, can, glasses, marker, ball, mobile phone, remote control
- R3: mobile phone, scissors, remote control, ball, light bulb, can, plug adapter, glasses, cup, marker
- R4: glasses, scissors, can, light bulb, cup, plug adapter, ball, mobile phone, remote control, marker

The same assignments as shown before (Tables 1, 2) are used for hyperparameter optimization (see Tables 3, 4). However, the activation threshold assignments are extended by three values: [0.01, 0.01], [0.05, 0.05], and [0.07, 0.07] (see Table 3) to reduce the node creation probability, which can lead to smaller GDM networks. The best assignments (bold letters) reveal that the GDM can benefit from having a smaller activation threshold (i.e., [0.05, 0.05] with 85.3% average accuracy). However, smaller values, like [0.01, 0.01], demonstrate only 39% average accuracy, indicating that even smaller values will show worse results and that we found a reliable assignment for this hyperparameter.

**Table 3:** This table shows the considered GDM hyperparameter assignments for the grid searches of the CORe50 experiment.

**Parameter**	**Description**	**Assignments**
*K*	No. of context vectors	**2**, 4
[ϵ_*b*_, ϵ_*n*_]	Learning rates	[0.1, 0.001], **[0.3, 0.003]**, [0.5, 0.005]
$\left[a_{th}^{E},a_{th}^{S}\right]$	Activation thresholds	[0.1, 0.01], [0.3, 0.03], [0.5, 0.05], [0.01, 0.01], **[0.05, 0.05]**, [0.07, 0.07]

*The best results averaged across all grid search iterations are shown in bold letters. For further descriptions of the parameters, see Table 1*.

**Table 4:** This table shows the considered A-SOINN+ hyperparameter assignments for the grid searches of the CORe50 experiment.

**Parameter**	**Description**	**Assignments**
*K*	No. of context vectors	**2**, 4
[ϵ_*b*_, ϵ_*n*_]	Learning rates	[0.3, 0.003], [0.5, 0.005], [1, 0.001], **[2, 0.001]**, [3, 0.002]

*The best results averaged across all grid search iterations are shown in bold letters. For further descriptions of the parameters, see Table 2*.

### 4.2. Results

**Figure 5:**
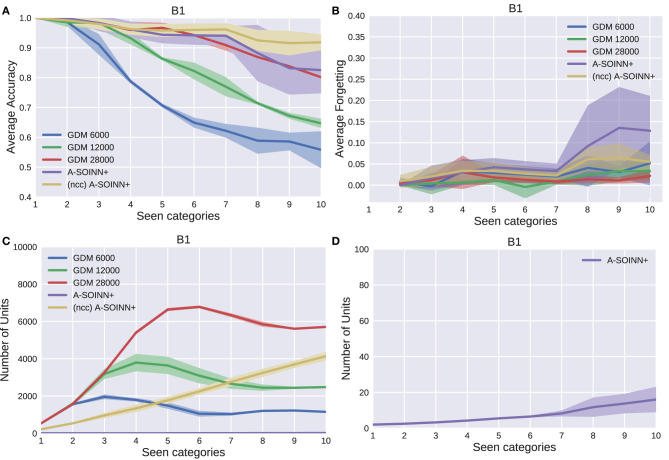
The results of the first experiment. GDM 6,000, GDM 12,000, GDM 28,000, A-SOINN+, and A-SOINN+ without node creation constraint are represented by blue, green, red, purple, and yellow lines. The x-axes represent the number of learned tasks and, therefore, the number of seen categories over time from 1 to 10. **(A)** is showing the average accuracy of each model on the y-axis in the interval 0.4 to 1.0. **(B)** is presenting the average forgetting in the interval −0.04 to 0.4, **(C)** the number of units in the interval 0 to 10,000, and **(D)** the number of units focused on the A-SOINN+ model in the interval zero to 100. All results are averaged across the four repetitions. The shaded areas represent the resulting standard deviation values.

**Figure 6:**
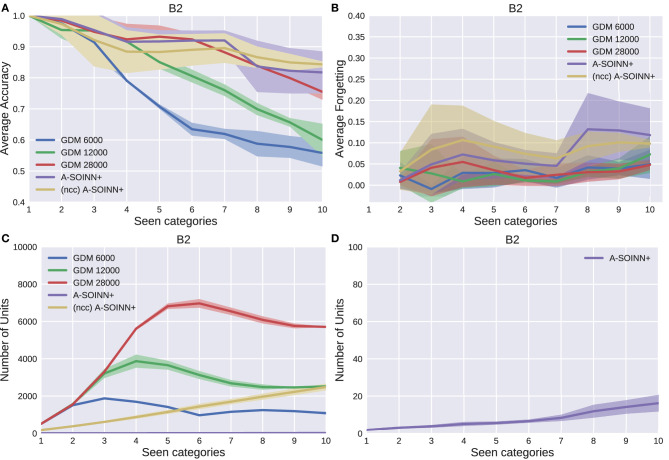
The results of the second experiment. For further descriptions, see Figure 5.

**Figure 7:**
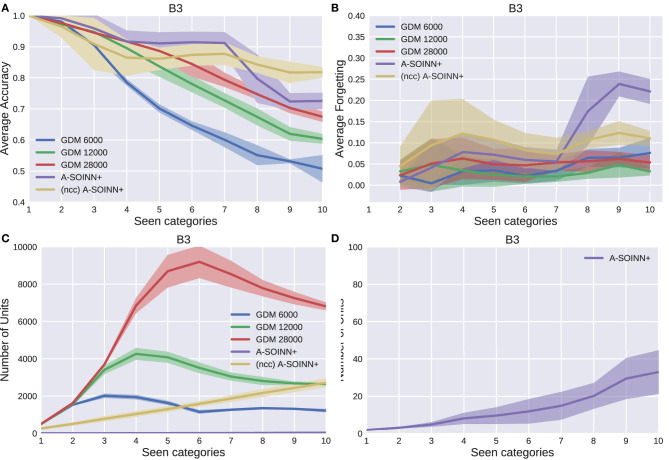
The results of the third experiment. For further descriptions, see Figure 5.

**Figure 8:**
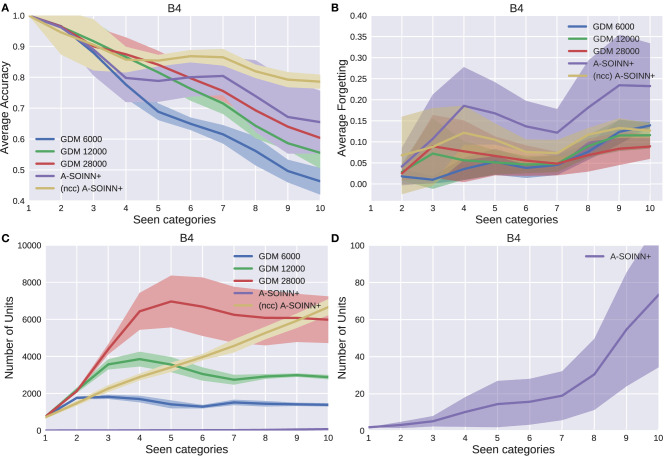
The results of the fourth experiment. For further descriptions, see Figure 5.

**Figure 9:**
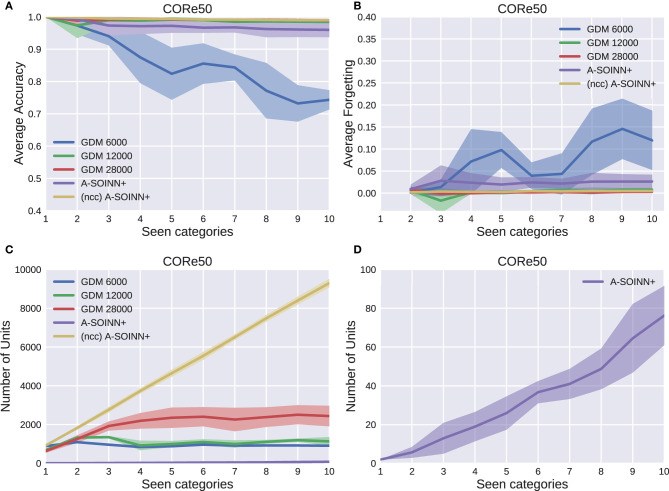
The results of the fifth experiment, using the CORe50 dataset. For further descriptions, see Figure 5.

Figures 5–9 show the results of all five experiments. In the presented graphs, the results of the models GDM 6,000, GDM 12,000, GDM 28,000, A-SOINN+, and (ncc)A-SOINN+ are shown in blue, green, red, purple, and yellow, respectively. The shaded areas represent the standard deviation created by computing the arithmetic mean across the four repetitions. The x-axes show the number of tasks already learned by the models, hence the seen categories. Sub-figures labeled with the letter A are showing the average accuracy results in the range 0.4 to 1.0, B the average forgetting results in the range −0.04 to 0.4, C the number of units of each model in the range 0–10, 000, and D the number of units focused on the A-SOINN+ in the range zero to 100.

#### v-NICO-World-LL Experiments

By considering Figures 5A, 6A, 7A, 8A, it can be observed that the average accuracy value generally decreases with an increasing amount of learned tasks, independent of the model or the background complexity. Additionally, the curves are lower for models trained on the two most complex experiments. Among the other GDM models, the GDM 28,000 reaches the highest average accuracy values, followed by GDM 12,000 and GDM 6,000. Hence, the higher the maximal age, the higher the average accuracy. In the first three experiments, the A-SOINN+ approach displays similar or even better average accuracy progress than the GDM 28,000. Between tasks three and five of the fourth and most challenging experiment, the accuracy results of the A-SOINN+ are lower than those of the GDM 12,000. The standard deviation values of the A-SOINN+ are higher in the experiments of *B*1, *B*2, and *B*4. Especially after learning task eight, an increase of the standard deviation is observable, accompanied by a decrease in the average accuracy. This behavior is not visible in the results of the GDM models. The A-SOINN+ without node creation constraint shows the highest average accuracies in the first and fourth experiment, together with a lower standard deviation than the A-SOINN+ with node creation constraint. After the last task, the (ncc)A-SOINN+ and the A-SOINN+ show the best average accuracy results in each experiment.

No substantial average forgetting (Figures 5B, 6B, 7B, 8B) increases are observable in the GDM model curves, especially not in the first three experiments. In the fourth one, the increase and the standard deviation are higher than in the other three experiments. However, the average forgetting is comparably low and does not exceed the value 0.17. In contrast to that, the average forgetting of the A-SOINN+ is higher in each experiment. It increases after the model learned task eight. This increase corresponds to the average accuracy drop after this task mentioned earlier. The (ncc)A-SOINN+ shows the second-highest forgetting curves. However, it has no substantial forgetting increase at task eight.

An essential part of this work is comparing each model's memory requirements, which are directly linked to the number of nodes created by the models. It is also related to the computational requirements since for BMU determination, an iteration over the whole network is required. In each experiment (Figures 5C, 6C, 7C, 8C), each GDM model's unit number reaches a maximal point, slightly declines, and converges afterward. The maximum unit number of the GDM 6,000 is approximately 2,000 in each experiment, and the value converges at approximately 1,000 units. In GDM 12,000, the maximal point is approximately 4,000 and converges at approximately 2,800. GDM 28,000 shows a maximal number of approximately 7,000 and converges at approximately 6,000 units in the first, second, and fourth experiment. However, the fourth experiment's standard deviation is higher than in the other. An exception is the third experiment, where the GDM 28,000 shows a maximal value of approximately 9,000, and no convergence can be observed until task 10. However, due to the other results, it is expected that convergence would take place with additional tasks. In general, a GDM model with a higher maximal age shows a larger number of units during LL. Additionally, it reaches the maximum number at a later point in time than GDM models with a lower maximal age. The (ncc)A-SOINN+ shows a linear unit number increase in all experiments. The maximum number at the end of the training is approximately 4,000, 2,200, 2,500, and 7,000 in the four experiments, respectively. Figures 5D, 6D, 7D, 8D focus on the unit number of the A-SOINN+ approach. The first and the second experiment show a maximal number of approximately 20 nodes with a comparably low standard deviation. The maximal number grows to approximately 30 in the third experiment, and the standard deviation increases. In the fourth experiment, the maximal number of nodes increases to approximately 70. Furthermore, the curve shows a generally higher standard deviation than in the other three experiments. In general, the A-SOINN+ is showing the lowest number of neurons. However, this number does not converge like in the GDM approaches. In the first three experiments, it grows roughly linear. In the fourth experiment, the slope increases over time, resulting in a stronger than linear behavior.

#### CORe50 Experiment

Figure 9A shows that except for the GDM 6,000, all models have a high and nearly constant average accuracy of at least 0.97. Similar behavior is observable in Figure 9B. The average forgetting of all models, except for GDM 6,000, is constantly lower than 0.06. As shown in Figure 9C, the number of units converges for the GDM approaches with a maximum of around 1, 000, 1, 500, and 2, 300 nodes for the GDM 6,000, GDM 12,000, and GDM 2,8000, respectively. The (ncc)A-SOINN+ unit number increases linearly and reaches a maximum value of approximately 9, 500. A linear unit number increase is also observable for the A-SOINN+ (Figure 9D). However, as in the previous experiments, the slope is much lower than in the (ncc)A-SOINN+ with a maximal value of approximately 75 units and, therefore, 30 times fewer than the best GDM model.

Table 5 is summarizing all five experiments. The average accuracy of each model is averaged across all 10 tasks. The A-SOINN+ approach achieves the highest values (gray shaded cells) either with or without the node creation constraint. The A-SOINN+ with node creation constraint shows either the best or second-best results in the first four experiments. In the CORe50 experiment, the GDM 12,000, GDM 28,000, and both A-SOINN+ models show similar high results with an average accuracy of 97.26% for the proposed model. Table 6 shows the training time (in minutes) and the file size (in megabytes) of the different models in the B4 experiment without considering the testing time. The models are trained on an Intel Core i7-4930 K CPU with 3.40 GHz. Due to the sequential nature of LL, we are not able to parallelize the training. The A-SOINN+ shows the lowest training time of around 4 min averaged across all four repetitions. This is followed by the GDM 6,000. GDM 12,000 and (ncc)A-SOINN+ show similar training times of 363 ± 58 and 370 ± 22 min, respectively. The GDM 28,000 exhibits the highest training time of 1,704 ± 437 min (≈28.4 ± 7.3 h). The lowest memory requirements of around 0.7 MB are observable in the A-SOINN+, followed by GDM 6,000, GDM 12,000, GDM 28,000, and the (ncc)A-SOINN+, which exhibits the highest memory requirements of around 741 MB.

**Table 5:** This table shows the arithmetic mean of the average accuracies across all 10 tasks (in percent) for each model (columns) in each experiment (rows).

**Dataset**	**GDM 6,000**	**GDM 12,000**	**GDM 28,000**	**A-SOINN+**	**(ncc)A-SOINN+**
B1	73.95 ± 1.78	83.88 ± 0.66	92.60 ± 1.31	93.07 ± 3.47	95.71 ± 1.83
B2	73.65 ± 1.68	81.95 ± 2.02	89.86 ± 2.21	90.7 ± 3.62	90.06 ± 2.94
B3	72.09 ± 1.58	80.56 ± 2.08	84.88 ± 1.73	88.51 ± 2.21	88.28 ± 2.00
B4	70.96 ± 2.86	78.25 ± 2.63	80.69 ± 2.74	81.07 ± 6.30	86.93 ± 1.54
CORe50	85.58 ± 3.02	98.73 ± 0.97	99.19 ± 0.46	97.26 ± 1.76	99.33 ± 0.30

*The gray shaded entries represent the highest values for each experiment*.

**Table 6:** This table shows the training time (in minutes) and the file size (in megabytes) of each model in the B4 experiment.

**Model**	**Training time (min)**	**File size (in MB)**
GDM 6,000	129 ± 25	39 ± 4
GDM 12,000	363 ± 58	146 ± 10
GDM 28,000	1704 ± 437	649 ± 226
A-SOINN+	4 ± 0.7	0.7 ± 0.4
(ncc)A-SOINN+	370 ± 22	741 ± 77

*The values are averaged across the four repetitions and shown together with the corresponding standard deviations. The gray shaded areas represent the lowest values*.

## 5. Discussion

Although the A-SOINN+ approach exhibits higher average forgetting curves, it reaches average accuracy values comparably high to the best GDM model. Additionally, it shows considerably lower memory requirements regarding the number of units. In the fourth experiment, the difference consists of approximately 28 times fewer units than the GDM 6,000 and approximately 100 times fewer than the GDM 28,000. Here, the maximum of each unit curve is used for comparison. In the first experiment (Figure 5), the highest difference between the two approaches is observable. The A-SOINN+ consists of around 100 and 350 times fewer nodes than the GDM 6,000 and GDM 28,000, respectively. Removing the node creation constraint can improve the performance of the A-SOINN+. However, it also increases the memory requirements since nodes are created even if the input has the same label as the prediction.

Nevertheless, the proposed model also shows drawbacks. It exhibits a generally higher standard deviation, especially after task eight in the v-NICO-World-LL experiments. Hence, the different category orders (repetitions) cause different performances. We call this effect *category order effect*. After analyzing each repetition of the first four experiments individually, we observe that especially R1 is causing poor results. However, sometimes R2 and R3 cause poor results as well. With a more in-depth analysis of the training procedures, we observe that as soon as the categories “pocket watch” and “present” become available, the performance decreases. In R1, R2, and R3, these objects become available at tasks 8 and 9, respectively. At the same time, the unit number for both categories increases. We further observe that the categories “present” and “pocket watch” are often misclassified as “ball” or “doughnut.” We assume that the categories' intra-category dissimilarity is large due to high differences among the corresponding instances. This dissimilarity and the mentioned misclassification as “ball” or “doughnut” can explain the higher number of units. Due to the comparably high number of “present” units and its similarity to existing neurons, the task-dependent test sets of previous similar categories are more likely to be misclassified as a “present,” resulting in low accuracy and high forgetting. As we never train the model on previous samples again, the nodes remain unchanged, and the performance stays low. We further assume that this category order effect is amplified with an increasing background complexity, resulting in a higher standard deviation. It is assumed that the inter-category distance is larger in higher background complexities due to a more substantial influence of the background on the feature vectors. We hypothesize that this effect occurs due to the small number of neurons in the network and because already trained neurons are never retrained afterward. Therefore, we suggest two extensions for future work: (1) removing the creation constraint or (2) implementing a (pseudo-)rehearsal technique. The first allows the creation of more units for one category, which can lead to a higher probability of being predicted correctly. The second can retrain existing neurons to represent the corresponding category better. Our improvement suggestions are supported by the results of the (ncc)A-SOINN+ and the GDM models. The former has no node creation constraint, and the latter use memory replay (pseudo-rehearsal), and in all four, the category order effect does not occur. However, the (ncc)A-SOINN+ shows an infeasible unit number increase. Therefore, we recommend incorporating (pseudo-)rehearsal in the A-SOINN+ for future work.

An essential aspect of LL is a high performance even after thousands or hundreds of thousands of tasks, including the memory requirements. In all five experiments, the unit number of the GDMs converges and increases linearly or stronger than linearly in the proposed model. In the CORe50 experiment, the A-SOINN+ would become less efficient than the best GDM after approximately 30 times more tasks (around task 310), assuming a naive continuation of the unit number curves and a constant average accuracy after task 10. In the v-NICO-World-LL experiments, this would happen after 200 times more tasks (around task 2,000) at the earliest. However, due to the decreasing average accuracy behavior in the first four experiments, such a prediction is unreliable. It is much more likely that the GDM will increase its neuron number or drop in performance. On the other hand, the A-SOINN+ can grow continually to learn a theoretically unlimited amount of tasks. However, this, in turn, can lead to infeasible memory requirements. Therefore, we recommend further investigations in this direction to evaluate the proposed model's long-term usability on a larger dataset, e.g., by expanding the v-NICO-World-LL with additional categories. Two further aspects of the dataset can be tackled in future work to create a higher real-world resemblance. The robot's arm movements are smooth to create sharp images, and jittery movements are not included. However, they take place in a real-world HRI scenario. Furthermore, the object's physical properties like weight or texture do not influence the arm movement in our scenario. Due to the adaptability of a virtual 3D scenario, jittery movements and physical object properties can be included in future work.

The results of our experiments demonstrate that for the two tested datasets, the pruning strategy of the original SOINN+ approach (Wiwatcharakoses and Berrar, 2019) and the adaptations made in this work lead to an efficient LL model for classification tasks in terms of average accuracy and memory requirements. The latter is observable in the unit number and in the file size of each model. We further observe that the training time of the A-SOINN+ is approximately 268 times lower than the one of the GDM 2,8000 in the fourth experiment. We attribute this to the fact that for BMU determination, an iteration over the whole network is required, making the computation time grow linearly with the number of units. Although the A-SOINN+ without creation constraint shows similar high unit numbers as the GDM 28,000, its training time is four times lower. We assume that GDM's memory replay is causing higher training time as additional iterations over the network are performed. These aspects indicate higher suitability of the A-SOINN+ for autonomous robots with limited computational resources.

## 6. Conclusion and Future Work

### 6.1. Conclusion

This work investigates whether the A-SOINN+ approach can reach state-of-the-art classification accuracy results compared to the GDM architecture while showing fewer memory requirements in terms of created neurons. Two main adaptations are made compared to the original SOINN+ (Wiwatcharakoses and Berrar, 2019). (1) An associative matrix is used that stores for each node a frequency-based distribution of input labels to enable classification, and (2) top-down cues to regulate the structural plasticity of the network are introduced in the form of additional constraints for node creation and weight adaptation. The models are tested on two LL object recognition datasets, namely CORe50 and the v-NICO-World-LL. The latter is a novel LL dataset proposed in this work. This dataset exhibits three novel features that are, to our knowledge, currently not considered as a whole by other LL object recognition datasets: (1) four different background groups of different levels of complexity are defined, (2) a virtual robot is manipulating objects instead of a human to simulate a long-term HRI scenario where the robot receives different objects over time, and (3) it is recorded in a nearly photorealistic virtual environment making it highly controlled. The dataset consists of 100 objects belonging to 10 categories. These categories could also appear in an HRI scenario. The results of five experiments show that the A-SOINN+ approach reaches high average accuracy results during LL and, in general, it is as accurate as the best GDM model. Furthermore, it requires fewer neurons than the other models, with at least approximately 30 and at most 350 times fewer units than the best GDM of each experiment. Additionally, its training time is approximately 268 times lower.

In summary, our contributions are:

1. The A-SOINN+ approach is developed, which is a novel and efficient version of an existing unsupervised LL approach for classification tasks.
2. A novel, nearly photorealistic LL object recognition dataset is created using a virtual humanoid robot. This dataset's main feature is the grouping of the environments into different levels of complexity. The dataset and methodology for synthetic data generation can be made available to the research community.

### 6.2. Future Work

Future work can tackle the long-term behavior of the A-SOINN+. In this context, the v-NICO-World-LL can be expanded with additional categories. A more in-depth analysis of different feature extractor architectures and data splits for transfer learning can also be examined in future works. Furthermore, it would be interesting to examine whether the proposed model can continuously learn from a multi-modal signal, like an audio-visual stream. Different aspects of a real-world HRI scenario are not considered in this work but can influence the performance of the A-SOINN+ and the long-term HRI experience in general. Grasping objects is not an easy task for real robots, and incorrect grasping can cause the object to slip out of the robot's hand. Furthermore, depending on the hardware, cameras might create blurred images due to jittery robot arm movements. Future work can examine whether the A-SOINN+ behaves differently on such images by applying it to a real robot. The results shown in this work indicate higher suitability of the A-SOINN+ approach for autonomous robots with limited computational resources. However, whether this applies to thousands or hundreds of thousands of tasks remains an open question. Nevertheless, this work is a further step toward social robots that continually acquire knowledge through long-term human-robot interactions.

## Data Availability Statement

The datasets presented in this article are not readily available because not all 3D models are licensed under CC BY. Requests to access the datasets should be directed to a.logacjov@gmail.com. We would like to publish a partial dataset, including all objects licensed under CC BY. Once available it will be placed on https://www.inf.uni-hamburg.de/en/inst/ab/wtm/research/corpora.html.

## Author Contributions

AL and MK conceived the presented idea. AL developed the neural architectures and conducted and evaluated the experiments with support from MK. AL was the primary contributor to the final version of the manuscript. SW and MK supervised the project and revised the manuscript. All authors provided critical feedback and helped to shape the research, analysis, and manuscript.

## Conflict of Interest

The authors declare that the research was conducted in the absence of any commercial or financial relationships that could be construed as a potential conflict of interest. The handling editor declared a shared affiliation with several of the authors AL, MK, and SW at time of review.

## Footnotes

^1^The Algorithm and all subroutines are created by following the explanations and notations of Wiwatcharakoses and Berrar (2019) and Parisi et al. (2017).

^2^The dataset is available at https://www.inf.uni-hamburg.de/en/inst/ab/wtm/research/corpora.html.
